# Reflecting on clinical education

**DOI:** 10.1007/s40037-013-0059-y

**Published:** 2013-04-19

**Authors:** B. Bonke

**Affiliations:** Erasmus University Medical Center Rotterdam, Rotterdam, the Netherlands

Welcome to this second issue of *Perspectives on Medical Education* in its second year of existence. As Editorial Board we are happy with the increasing number of manuscripts that have been submitted in the past 18 months or so, many of which are important for the field of medical education, and we look forward to many more years to come. Let me introduce you to the content of the *PME* issue #2, where feedback and reflection in the professional workplace take a prominent role.

Alexandra Hay is a fifth-year student at Manchester Medical School, UK, and the first author of a paper entitled *Medical students’ reactions to an experience*-*based learning (ExBL) model of clinical education* [1]. She tested the ExBL model in a group of 19 volunteering junior clinical students who discussed how the model corresponded to their experiences of clinical learning. Qualitative analysis was carried out on the verbatims of group discussions and on 500-word reflections that the participants wrote some weeks later. In these reflections the participating young doctors considered their clinical experiences before and/or after the group discussion, linking them to ExBL. Hay’s results were consistent with a strong effect of environment on learning processes and she and her co-workers rightly emphasize the important role of affective support for the learner. The Editorial Board of *PME* is proud to serve as a platform for such young scientists and to offer the fruits of Alexandra Hay and her co-workers to its readers.

Another way for students to explore their perspectives and possibilities is to travel. Fortunately enough, medical students often get the opportunity to go and study abroad. What a chance to sharpen their focus and learn that medicine and health are distributed so differently. Many look for a country in a different continent and/or a health institute where the medical profession offers them the chance to increase their knowledge and skills. Some travel to carry out research, or do a PhD, others leave their country of origin to take their clinical rotations abroad or even run a local hospital in rural Africa. Two medical students from Saudi Arabia, Ahmad Adi and Hani Alturkman from Alfaisal University in Riyadh, crossed the water to interview Dr. John E. Hall, i.e., the author of the ‘Guyton and Hall Textbook of Medical Physiology’ [2]. Obviously eager to become successful doctors they learned that the ‘secret’ to success is to find something that you really love, and if you love it, it’s not work. Yes, it can be as simple as that. Or is it? What if you have a mobile device that allows you to combine your love for the profession with your fondness of the internet and e-learning? Sounds fun!

Sylvia Eggermont and her co-authors describe the use of the portal MedicalEducation.nl on a small screen at Leiden University in the Netherlands and noticed a substantial increase in this use of mobile devices among students and much less so among medical professionals [3]. The authors see a brighter world on the horizon if and when the use of e-learning on mobile devices is better supported, but would empathy increase if doctors relied heavily on the internet? Where is the old-fashioned role model? If lectures and bedside teaching are replaced by electronic devices, and webinars or other pre-recorded talks from distinguished professionals take the place of the person-to-person feedback, advice, encouragement, or correction, then our medical schools will have to find other ways to ensure the development of professional behaviour, emotional responses and empathy.

Can one indeed measure the teaching methods of supervisors during residency training? This question was addressed by Lia Fluit and her co-authors in their paper *Repeated evaluations of the quality of clinical teaching by residents* [4]. They evaluated supervisors of three medical specialities, using the EFFECT, a validated instrument measuring 11 skills, e.g., role modelling skills, feedback content, assessment, and personal support. The authors investigated, in two subsequent years, whether resident ratings of 24 clinical teachers improved after this evaluative strategy and what degree of improvement, if any, they detected. Their results show a moderate effect, with more pronounced increases in those clinical teachers who initially scored relatively low. What is striking, though, is the high average scores on the questionnaire, with more than half of the clinical teachers scoring >4.5 on a 1–5 rating scale. That is, halfway between ‘satisfactory’ and ‘good’. Do we notice a ceiling effect? In my opinion, labelling of rating scores with adjectives is subject to interpretation differences and although this may lead to an ordinal scale, the intervals of such a scale in a questionnaire are not necessarily equal. Besides, who, as a resident, dares to rate a clinical teacher as ‘poor’ (score 2) or even ‘very poor’ (score 1)? Could the use of a visual analogue scale, instead of the scale used, perhaps help to avoid ceiling effects? And, what is the objective quality of clinical teachers? As Kimberly McLaren et al. [5] put it in their article *ownership of patient care: a behavioural definition and stepwise approach to diagnosing problems in trainees*: ‘as graduate medical education evolves, teaching faculty are tasked with balancing supervision requirements with trainee autonomy, all the while ensuring patient safety and quality of care’. Certainly not an easy job! When does a trainee show adequate ownership of patient care? McLaren and her colleagues come up with a behavioural definition of patient ownership: advocacy, autonomy, commitment, communication, follow-through, knowledge and teamwork. Their consensus-derived definition provides helpful tools for clinical teachers to evaluate trainees systematically and objectively. And also to determine any particular deficits and find out how these can be remediated in the practice environment.

But perhaps the trainees that you as a reader are involved with generally fulfil the quality goals set for them? Yet, providing relevant feedback as a clinical teacher and stimulating thorough reflection by the trainees, based on observed performance in the workplace, is difficult, time consuming, and all the while highly important. The work that Elisabeth Pelgrim’s and her co-authors publish in a Letter in today’s *PME* issue focuses on feedback, reflection, and the development of action plans in various general practices in Nijmegen, the Netherlands [6]. Giving feedback can lead to the formulation of an action plan, but this is much more likely if attention is also paid to the trainee’s reflections. Lastly, if you want to learn why a British educator of evidence-based medicine advises us to rename our curricula as knowledge translation curricula, then read Iman Hassan’s Letter [7] and perhaps respond through *PME*? You are very welcome!

Reflection as a learning tool independent of feedback needs to be nurtured, and this is exactly what this *PME* issue intends to offer you: new perspectives to reflect upon. The Editorial Board of *PME*, recently enriched with prominent colleagues from various countries, intends to push *PME* forward into becoming a leading journal in the field of medical education.
